# On the Application of the Ligature to Wounded Arteries, or Their Trunks, at a Distance from the Wounded Part and Nearer the Heart. An Essay on the Treatment of Traumatic Hemorrhages

**Published:** 1837-07

**Authors:** 


### Art. X.

*Ueber die anwendung der Ligatur an einer von der Wunde entfernten, dem herzen zugewendeten stelle der verwundeten Arterie oder des entsprechenden Arterienstammes. Ein Beitrag zur Therapie der traumatischen Blutungen von* Karl Joseph Beck, m.d., Professor der Chirurgie, &c. in Freiburg, &c.—*Freiburg*, 1836.

*On the Application of the Ligature to Wounded Arteries, or their Trunks, at a Distance from the wounded Part and nearer the Heart. An Essay on the Treatment of Traumatic Hemorrhages.* By K. J. Beck, m.d., Professor of Surgery, &c. at Freiburg, &c.—*Freiburg*, 1836. 8vo. pp. 79.

The readers of this little work will find that it has no pretensions to novelty, either as regards principles or practice; nor, indeed, does the author claim any such distinction for it himself. It is, nevertheless, well worthy the perusal of the surgeon; since, among the cases here enumerated, he will find a precedent for almost every variety of wounded artery which can possibly occur to him in his practice; and the reasons which are given for preferring certain modes of treatment in certain cases, are at once sound, judicious, and obvious to the understanding. They involve no new theory, but are in strict accordance with well known and acknowledged principles of surgery. When the result of experience is thus brought to bear upon principles respecting which there can be no dispute, rules may safely be established which are entitled to our confidence, and which may be followed with every reasonable anticipation of success. If we have any fault to find, it is with the general tenour of the volume, and the strong predilection which the author has evidently conceived in favour of one particular scheme of practice, which, although it has its advantages, and is often indispensable, yet is not without its dangers; and to these he makes no allusion. We shall lay before our readers a brief analysis of Dr. Beck's book, confining ourselves within those limits indicated by the title, viz. the arrest of arterial hemorrhage by ligature.

The views and object of the Doctor's enquiry are thus stated by him.

"The mode of arresting hemorrhage, under certain circumstances, may be considered as one of the most difficult problems which science is called upon to solve. The solution is, indeed, easy when the bleeding vessel is superficial; when the hemorrhage is evidently caused by the mechanical injury and lesion; and when there exists neither physical nor structural deviation from the healthy normal state, either as regards the vessel or the surrounding parts. But, when the source of hemorrhage is so deeply seated that it can neither be ascertained with certainty nor brought into view; when the depth of the injured parts and the importance of the superjacent structures render a direct exposition either arduous or impossible; in such a case are we likely to find ourselves embarrassed by the difficulties which present themselves, and the uncertainty of the result. The operation necessary to lay bare the wounded vessel may be highly injurious, and the difficulties to be anticipated in the execution of our object may excite such well-grounded fears for the safety of the parts,—even for the life of the patient,—as to render the project of a direct exposition untenable. Thus, under circumstances when death can only be averted by prompt, judicious, and decisive measures, it may happen, from the peculiarities of the case, that the surgeon finds himself involved in doubt as to whether he shall secure the vessel at the wounded part, or apply his ligature at some point above the seat of injury, i. e. nearer the heart; or else tie the parent trunk from which the wounded branch is given off. On this subject I shall endeavour to lay down decided rules for practice, founded on my own experience as well as that of others." (P. 1.)

Two modes, therefore, of arresting hemorrhage by the application of a ligature present themselves to the surgeon. He may secure the artery at the seat of injury, in which case a ligature should be placed above and below the opening in the vessel; or he may tie the artery, or else the parent trunk, at a distance from the original wound. The first of these methods may be called the *local*, the second the *remote*, application of the ligature. To regulate his choice in the adoption of one of these two methods, under existing circumstances, is the object of Dr. Beck's enquiry.

The application of the local ligature is doubtless the most obvious, the safest as regards the result, and therefore the best; but there are many cases in which collateral circumstances render its adoption inadmissible, and then the remote ligature must necessarily be had recourse to.

"We have both authority and experience to justify us in considering the remote ligature as an efficient means of arresting hemorrhage. Under certain circumstances, it presents the only method which can be adopted, and in some rare instances it is even preferable to the local ligature." (P. 16.)

We will now proceed to enumerate what these various circumstances and conditions are, which, in Dr. Beck's opinion, render the application of the remote ligature advisable, if not necessary: or, in other words, which render it impolitic or impracticable to secure the artery at the wounded spot.

They are arranged in the following order:

"*Rule* 1*st.* When the vessel which is apparently the source of hemorrhage is so deeply situated and concealed as to preclude the probability of its being effectually exposed: we may also add those cases of secondary bleeding, where, either because the process of healing is already advanced, or where, from any other circumstance, it does not appear expedient to reopen the original wound." (P. 24.)

Under this head are included those cases where the exact source of the hemorrhage cannot be readily ascertained; when the deep seated mischief does not correspond in situation with the external wound, and the direction of the latter is uncertain; where the wounded artery is placed between or in the neighbourhood of bones which it would be inexpedient to lay bare; or when the superjacent parts are of too great importance to risk their disturbance or division.

A number of cases are *detailed* to illustrate the above rule. We abridge the following, which occurred in the author's own practice. The first affords a useful example of bad surgery.

Case i. A young man received a sword-wound in the middle of the thigh, followed by profuse arterial hemorrhage, and accompanied by internal effusion of blood. It was impossible to ascertain whether the femoral or profunda artery, or both, had suffered. The wound was enlarged and explored, but without success. The femoral artery was then cut down upon, and secured, in the ordinary manner, at the junction of the upper with the middle third of the thigh. The next day the bleeding returned, and the whole limb had now become immensely swollen, besides being in a state of acute inflammation. A second ligature was applied round the femoral at Poupart's ligament; but, although this proved effectual in preventing the recurrence of hemorrhage, the patient sunk exhausted, and died on the third day. We must, in justice to Dr. Beck, observe, that, although he was the operator in this mismanaged case, he acted under the control of higher authority, and contrary to his own expressed judgment. The two official personages, bearing the titles of Gerichtsärtz, and Geheimer Hofrath, must share the blame between them.

Case ii. A boy, seven years old, ran a penknife into his thigh, which apparently pierced the femoral artery just as it enters the popliteal space: the blood gushed from the wound, and considerable swelling from internal effusion soon manifested itself in the ham. Pressure at the groin commanded the hemorrhage. The femoral artery was tied in the usual situation with complete success.

Case iii. A man, aged twenty-seven, received the contents of a gun, loaded with large shot, in the back of his thigh, inflicting twenty-eight wounds between the buttock and the knee: three of the shot had traversed the limb, and passed out on the fore part. Much blood was lost at the time, and the limb became swollen. Five days afterwards, a small fluctuating and pulsating tumour made its appearance on the anterior part of the thigh, at the junction of the middle with the lower third, and in the situation of the femoral artery. This was treated by pressure, but continued to enlarge; and, in five days more, ulceration of the skin took place, followed by considerable hemorrhage. As the site of the swelling indicated that the femoral artery itself, or an important branch, was wounded, the former was exposed, and tied just above the middle of the thigh: the tumour was then laid open, and the coagulated blood removed. The case terminated successfully.

Many other cases, collected from the best authorities, are also brought forward by Dr. Beck, and tend greatly to establish the validity of the rule which he has laid down. It occasionally happens that hemorrhage supervenes, unexpectedly, several days after the receipt of the injury, and when the granulating process has completely altered the original character of the wound, and afforded a well grounded anticipation of safe and speedy recovery. In such cases, the remote ligature is our only resource; and Dr. B. brings forward several instances in which it was successfully applied. We have ourselves seen the femoral artery tied for the purpose of arresting secondary hemorrhage from the stump of an amputated limb. The success was perfect; and we have seen the same treatment, with a similar result, adopted in cases of simple and compound fracture, which were followed by the formation of aneurism in the one instance and by external bleeding in the other. We have no hesitation in saying that, when some days have elapsed after amputation, it will generally be found far preferable to use the remote ligature, than to reopen the flaps of the stump, and search for the wounded vessel.

*Rule 2d.* "The remote ligature becomes necessary when such an altered condition of vitality and structure has taken place at the seat of injury as to render the local application of the ligature unsafe." (P. 24.)

It frequently happens that the primary hemorrhage from a wounded artery is repressed by pressure; the wound and the surrounding parts become filled with a temporary coagulum, which is effectual for some time in closing the opening of the vessel. Inflammation, however, supervenes; suppuration, or perhaps partial gangrene, according to the nature of the injury, become established: the clots separate, the artery is laid bare, and secondary bleeding ensues. Under these circumstances, Dr. Beck strongly reprobates the attempt to secure the vessel at the original seat of injury; not only because great difficulties will be experienced in the operation, but chiefly because the coats of the artery will have participated in the surrounding disease, will have become altered in structure, and lost the capability of taking on that healthy action, which, under the application of the ligature, is necessary for the sealing up and obliteration of the vessel. Several cases are cited to shew the advantages and success of the remote ligature under circumstances of this description. The same rule will likewise apply in those cases where an artery becomes opened by a process of ulceration, in consequence of malignant disease, abscess, or necrosed bone.

*Rule 3d.* "The remote ligature is generally necessary where the injury has involved the entire destruction of the artery." (P. 24.)

We do not think that the author has rendered his subject clearer or more explicit by laying down this rule. By the term *destroyed* (zerstört,) we presume is meant the entire removal or total disorganization of a portion of the cylinder of the vessel. Now, unless the artery has become exposed by the original injury, it is impossible for the surgeon to ascertain whether it has become completely destroyed or not; and thus the circumstances under which he is placed differ little from those which have already been discussed. Should the original injury present the artery to our view, torn through, bruised, or lacerated, it would certainly be advisable to follow the exposed extremities of the vessel from the wound, until we reached a spot where its walls were sound, and the ligature might be safely applied. If, however, such a proceeding should be considered as incompatible with the safety of the surrounding textures, then the remote ligature must be had recourse to.

*Rule 4th.* "When a considerable number of arteries are wounded, it may be necessary to apply the remote ligature to the common trunk from which they arise." (P. 24.)

A number of cases are brought forward in illustration of this position, such as punctured or incised wounds about the upper part of the neck, penetrating under the jaw, and involving the branches of the external carotid. In these the common carotid appears to have been tied with a successful result. Under this head are likewise included wounds of the palm of the hand, dividing the palmar arch, or the branches which it gives off. These are by far the most interesting to the surgeon, as they are accidents of constant occurrence, and often occasion not a little embarrassment. The communication is so free in the hand as to render it highly advisable that the vessels should be secured at the injured parts, in order that both ends may be tied; and certainly, when the injury is recent, where the wound is extensive, the palmar fascia freely divided, and the parts beneath exposed to view, an attempt to discover the source of hemorrhage ought to be made. The parts, however, are seldom in this favorable condition. A small, penetrating wound has generally passed under the fascia; little or no help is afforded for ascertaining its direction, and the hollow of the hand is most commonly filled and distended with effused blood. It frequently happens that some days have elapsed since the injury was received, and we find the whole hand swollen, œdematous, inflamed, and exquisitely painful. The patient has become exhausted by repeated losses of blood, and is suffering under a high degree of nervous irritation; all the symptoms, local and constitutional, having been aggravated by the application of styptics and powerful pressure for the purpose of checking the bleeding. Under these circumstances, there is but one resource,—that of tying either the radial or ulnar trunk, after having ascertained, by pressing alternately on each, from which of the two the hemorrhage proceeds. It may be necessary to secure both vessels. Dr. Beck seems to be altogether averse to local explorations in the palms, and instances several cases in which the ulnar artery was tied with success. Wounds of the plantar vessels or the arch in the sole of the foot are of very rare occurrence, and would hardly admit of any other mode of treatment than that of securing one or both of the tibial vessels.

*Rule 5th.* "The wound or division of an arterial branch, close to its origin from the parent trunk, may render it necessary to tie the latter." (P. 24.)

Case vi. A young man received a wound on the inner side of the thigh from a broken glass bottle, which divided the sartorius muscle and extended itself as high as Poupart's ligament. Copious hemorrhage followed: the bleeding vessel could neither be detected nor laid hold of by the forceps, and the external iliac trunk was consequently tied. The patient died from pneumonia on the thirteenth day; and it was then ascertained that the external pudic branch of the femoral had been divided close to its origin. It is, therefore, evident that in this case the treatment of the surgeon was not founded on his knowledge of the precise nature of the injury. The original wound had evidently involved either the femoral or some of its branches, and he preferred tying the iliac trunk to making any farther search for the bleeding vessel. In this, and in some other parts of his book, Dr. Beck has somewhat complicated his precepts by the unnecessary subdivisions which he has pursued in their arrangement.

*Rule 6th.* "When internal bleeding takes place, in consequence of injury to an artery, unaccompanied by external wound, or when fracture becomes complicated with a similar occurrence, recourse should be had to the remote ligature." (P. 24.)

The first of these conditions belongs more properly to the history and treatment of aneurismal formations; the latter has already been noticed in a former part of our review.

We extract the concluding paragraph of the Doctor's book:

"The operation which has formed the subject of this treatise is generally followed by a surprisingly speedy change in the condition of the patient, which must prove very gratifying to the humane feelings of the surgeon. No sooner has the ligature been applied, than the mind of the sufferer, which had been kept in a continued state of alarm and distress by the repeated hemorrhagies, becomes tranquillized. After the artery has been secured, the forcible compresses which were previously necessary can be exchanged for light and simple dressings. The pain, which was occasioned partly by the pressure, partly by the retained secretion from the wound, becomes diminished, inflammation is abated, a healthy suppurative action is established, and the gangrene is arrested; the patient begins to recover his powers, so soon as he is placed beyond the danger of future hemorrhage, and speedily loses that blanched appearance which was indicative of a deficiency of blood in the system." (P. 78.)

We have thus laid before our readers an abstract of Dr. Beck's essay, and have no hesitation in saying that he is entitled to the thanks of the profession for having, with considerable labour, brought together a number of highly useful facts and cases. Nevertheless, the sum of all that has been written and said on the subject of wounded arteries seems to resolve itself into this simple surgical canon.

In all serious cases of wounded artery, the application of a double ligature at the seat of injury must be considered (theoretically,) as the safest and most efficient treatment; but, where this mode of proceeding is rendered inadmissible from the various circumstances and conditions which Dr. B. has enumerated, then the application of the ligature must be transferred to some spot between the wound and the heart, the surgeon having previously ascertained, with all possible accuracy, whether pressure on that part of the vessel fixed upon for the operation be efficient in commanding the hemorrhage. It is clear, therefore, that no definite rule can be laid down. A wounded artery may, in one instance, be easily laid bare and secured; while, in another instance, a wound of the same artery, in the same spot, may, from collateral circumstances, lead us to anticipate so many difficulties and subsequent dangers in attempting to expose the seat of injury, as shall induce us to adopt the more indirect method of the remote ligature. The surgeon must therefore be guided by his own judgment and discretion; but promptness and decision are indispensable. To protect his patient from a recurrence of hemorrhage should be his immediate object. The adoption of half measures and a palliative mode of treatment are inevitably followed by two evils, viz. the exhaustion attendant upon repeated bleedings, and the mischief resulting from extensive internal effusion. These conditions will react upon each other, to the detriment or destruction of the patient; for, in proportion as his powers are reduced, so will the probability of a successful result from an operation become diminished; while, at the same time, he is rendered less capable of undergoing the extensive suppurative process often necessary to get rid of the effused coagulum.

We must now bestow a few words of honest and impartial criticism on Dr. Beck's opinions, and also gently deprecate what we consider to be the general tenour of the book, and the impression which it is likely to produce on the mind of the inexperienced reader; not that we at all wish to impugn the truth of the individual maxims which it contains, but because we fear that the zeal with which he advocates the principle of the remote ligature has caused him somewhat to outstep the bounds of discretion in recommending its too indiscriminate adoption. Throughout the whole volume, the author evidently betrays his decided preference for securing the artery at a distance from the wounded spot. In every case of difficulty, he proceeds at once to the fountain head. To an accomplished anatomist and an expert surgeon, such a mode of treatment certainly presents great attractions, and offers a temptation which it may be difficult to resist; but we question whether Dr. Beck does not ride his favorite hobby a little too hard: we doubt whether the operation which he advocates is so infallible or so universally successful as he represents it. He has certainly illustrated his opinions, and strengthened his positions, by a great number of successful cases, collated with much labour, and selected with considerable ingenuity; but we feel almost authorized to impute to him a certain want of candour, when he abstains from mentioning those unfortunate results which he must have met with in the course of his researches. We will not allude to the severity of a capital operation, (such as laying bare and securing a main artery,) nor the degree of risk attendant on it; but we would ask, is there no chance of gangrene to be anticipated from thus suddenly cutting off the supply of blood to an entire limb? Dr. Beck never so much as hints at the possibility of such a contingency, and yet we know that it does sometimes occur. We are perfectly aware that there are many cases in which we must incur the liability of future mischief to secure our patient from immediate destruction; but, at the same time, the surgeon should be made duly sensible of the responsibility which he takes upon himself, when he determines to turn the cock upon the main supplying a whole district, merely because one of the household service pipes has given way. We might also add that he must not consider himself already out of the wood, so soon as he has discovered the main road from which all the smaller paths divaricate. The very means which he has taken to accomplish his object of present security may not only prove the source of a fresh evil, but it may likewise altogether fail in the fulfilment of its original intention. He has broken the wheel at the fountain, the pitcher at the well; and the very treatment which he has pursued may prove mortifying (if we may be excused the term,) to his patient's limb, as well as to his own anticipations of success; while, on the other hand, the speedy re-establishment of collateral circulation, although it removes the danger of gangrene, may render his operation nugatory by affording a recurrence of the original hemorrhage. We have made these observations not with a view of opposing or controverting Dr. Beck's statements, but to shew that the application of the distant ligature does not at once place the patient beyond the pale of all possible farther mischief; that it should not be had recourse to on light and insufficient grounds. We would wish to shake that perfect confidence in its infallibility with which this book is calculated to inspire the mind of the young surgeon.

---

